# Pilot test of an online training module on near-infrared spectroscopy monitoring for the randomised clinical trial SafeBoosC-III

**DOI:** 10.1186/s13063-020-4206-6

**Published:** 2020-04-23

**Authors:** Mathias Lühr Hansen, Marie Isabel Rasmussen, Snorre Rubin, Adelina Pellicer, Guoqiang Cheng, Xin Xu, Yin Zhaoqing, Vibeke Zoffmann, Gorm Greisen

**Affiliations:** 1 Department of Neonatology, Rigshospitalet, Blegdamsvej 9, 2100 Copenhagen, Denmark; 2 Section for Learning Technology, Center for HR, Kildegårdsvej 28, 2900 Hellerup, Capital Region of Denmark Denmark; 3 Department of Neonatology, La Paz University Hospital, Paseo de La Castellana 261, 28046 Madrid, Spain; 4 Department of Neonatology, Children’s Hospital of Fudan University, 399 Wanyuan Rd, Minhang Qu, Shanghai Shi China; 5 Department of Neonatology, Key laboratory of Neonatal Diseases, Xiamen Children’s Hospital, 10 Zhenhai Road, Xiamen, Fujian province China; 6 Department of Neonatology, Dehong People’s Hospital of Yunnan Province, 13 Yong Han Street, Dehong Mangshi City, Yunnan province China; 7 Copenhagen University the department of Public Health, Section for Health Services Research, Rigshospitalet, JMC, Department of Women’s and Children’s Health, Tagensvej 22, 2200 Copenhagen, Denmark

**Keywords:** SafeBoosC, Randomised clinical trial, Randomized clinical trial, RCT, Extremely preterm, Near-infrared spectroscopy, NIRS, Online training, Web-based training, E-learning

## Abstract

**Background:**

SafeBoosC-III is an international randomised clinical trial to evaluate the effect of treatment of extremely preterm infants during the first 3 days of life based on cerebral near-infrared spectroscopy (NIRS) monitoring versus treatment and monitoring as usual. To ensure high quality of the trial intervention as well as of patient care, we have developed a multilingual web-based training program to train relevant staff and test their competence. As we enter an under-explored area of e-learning, we have conducted a pilot study on the first of the five modules comprising the web-based training program to test the feasibility of developing such a program for an international trial with limited resources.

**Methods:**

The module in this study focuses on the principles and practice of NIRS monitoring. The pedagogical idea was to integrate training and certification. One-hundred doctors and nurses from five Neonatal Intensive Care Units across China, Spain and Denmark were invited to participate in the pilot study. Upon completion of the NIRS module, participants were invited to evaluate their experience by completing an online survey. Data from closed-ended questions were analysed using descriptive statistics while data from open-ended questions underwent thematic analysis.

**Results:**

In total, 81 of 100 invited staff members entered the training module and completed the online survey. The median time and the number of questions to pass the module was 15 minutes and seven questions, respectively. Most staff found the academic level of the learning material and quiz appropriate (85% and 93% of all staff members, respectively), as well as agreeing that the module was relevant to prepare them to ‘use the NIRS device’ (90%). Thematic analysis revealed issues such as a discrepancy between learning material and quiz questions, lack of clarity, and technical issues.

**Conclusion:**

We provide evidence of the feasibility of developing a multilingual web-based training program for an international trial, despite challenges such as low budget, language barriers and possibly differences in the clinical training of staff. Exploring the integration of training and certification for international trials, the positive results of this study motivate further developments.

**Trial registration:**

ClinicalTrial.gov, NCT03770741. Registered 10 December 2018.

## Background

Randomised clinical trials are considered the highest level of evidence when evaluating the effects of a clinical intervention [1]. It is therefore essential that the methodological quality is high. Furthermore, since randomised clinical trials are conducted on human subjects [2], the safety and well-being of participants are of crucial importance. Good Clinical Practice (GCP) is an international standard for designing, conducting, recording and reporting clinical trials involving human subjects, with the purpose of ensuring the safety and well-being of trial subjects as well as high scientific quality [3]. A core principle in GCP is that staff members involved in the trial “should be qualified by education, training and experience to perform his or her respective task(s)” [3]. One way to ensure this is by training the clinical staff [4]. Despite evidence suggesting that training staff members in trial-related tasks has a positive effect on the trial’s results [5], the training process is rarely reported [6]. Furthermore, recommendations for specific training requirements for clinical trials are not defined in the standards on GCP by the International Committee on Harmonisation.

To recruit enough participants, large-scale clinical trials often include many centres across multiple countries. This poses the problem of training staff since on-site training is expensive, time-demanding and difficult to standardise. A way to bypass this issue, while preserving the quality of training, is by using e-learning. E-learning is a broad concept describing education facilitated through electronic systems, such as computers or mobile devices [7], and, as such, can be used to ensure standardised delivery of subject matter [8].

E-learning has already proven to be a valuable asset when increasing the competencies of health professionals, in both industrial and developing countries [8–13], and has been proliferating until now with the purpose of medical education at universities, as a counteraction to traditional classroom teachings [14]. It has proven to be a useful tool when harmonizing teachings that are aimed worldwide, across different languages and clinical settings, and has been used with great progress in resource-constrained countries [15].

A recent Cochrane review of e-learning, which it defined as any educational intervention mediated electronically via the internet, was found non-inferior to traditional classroom teaching [8, 16], and in the few published reports on e-learning as preparation for clinical trials, it has been implemented with success [4].

A consensus on a clear definition of e-learning does not exist. Therefore, multiple terms are used as synonyms for e-learning, including internet-based learning, web-based learning and training, computer-assisted instructions and computer-based learning and training [8, 17–19]. For the purpose of this study, we will use the term ‘web-based training’ (see the ‘Web-based training and certification program’ section).

### The SafeBoosC-III trial

SafeBoosC-III is a randomised clinical trial investigating the benefits and harms of treatment based on cerebral near-infrared spectroscopy (NIRS). The hypothesis is that treatment based on NIRS monitoring during the first 72 h of life of extremely preterm infants will result in a reduction of severe brain injury and death at 36 weeks postmenstrual age. Sixteen-hundred infants born with a gestational age below 28 weeks and admitted to more than 50 neonatal intensive care units across 20 different countries will be randomised. Infants in the experimental group will receive treatment guided by cerebral NIRS monitoring during the first 72 h of life, while infants in the control group will receive treatment and monitoring as usual. The protocol of the SafeBoosC-III trial is registered at www.ClinicalTrials.gov, NCT 03770741 (07.12.2018).

When working at a clinical department, it is often expected that you are familiar with routine practices. If you are not, learning will often happen through supervision by more experienced colleagues familiar with the interventions. However, when an intervention trial is rolled out in a clinical department, only a few staff members may be familiar with the intervention. Thus, training in trial-related procedures is necessary, not only for the safety of trial participants but also to give a relevant and practical estimate on the effect of the intervention in routine practice. This is done through a pragmatic trial such as SafeBooSC-III, where the purpose is to test the effect of a given intervention in a real-world setting [20], i.e. what effect can be expected by implementing a specific intervention in a broad patient group, in a large number of departments. Therefore, in order to estimate the potential effect of implementing an intervention in routine practice, staff on-site should be trained in the intervention before the trial takes off [3, 20]. However, if the trial ought to reflect how the intervention works in a real-world scenario, the level and intensity of staff training should reflect this, meaning that you do not want to train your staff members to an expert level prior to trial initiation since this does not reflect a ‘real-world’ scenario and you will not thus get generalizable trial results. To provide a practically realistic level of introduction and training for the SafeBoosC-III trial, we have developed a multilingual online training program to train relevant staff and test their competence.

As we enter an under-explored area of e-learning, we have conducted a pilot study on the first of the five modules comprising the web-based training program to test the feasibility of developing such a program on limited resources for an international trial. We expected that results from this pilot study could be used to enhance and support further development of the web-based training and certification program for SafeBoosC-III, and possibly encourage the use of e-learning when implementing future international clinical trials.

## Methods

Fifty nurses and 50 doctors from a total of five neonatal intensive care units across China, Denmark and Spain were invited to participate in the pilot study. In order to ensure that the e-learning tool was appropriate for staff of all levels of experience, the responsible investigators within each of the five participating neonatal intensive care units (AP, GC, MLH, XX, ZY) invited staff members with and without prior NIRS experience to participate. All neonatal intensive care units participating in this pilot study are planning to participate in SafeBoosC-III, and all participating staff members are expected to care for babies enrolled in SafeBoosC-III.

Participants were asked to 1) complete the web-based training module on NIRS monitoring and 2) evaluate it through an online survey.

### Web-based training and certification program

**Fig. 1:**
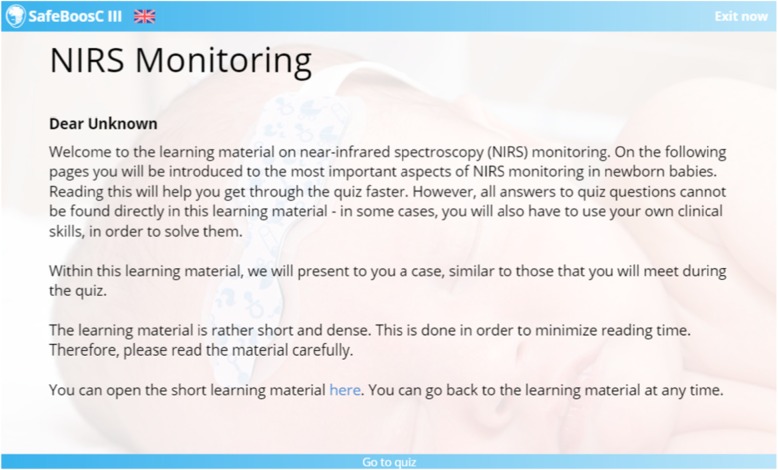
On the opening page, the participant will see a short introduction text and the possibility to 1) open the introduction material (‘*here*’ in *blue text*) or 2) go to the quiz (in the middle of the *lower blue bar*)

The training module is part of a complete web-based training and certification program for the SafeBoosC-III trial, which will be offered to all doctors and nurses involved in the care of trial participants. It consists of five separate modules covering 1) introduction to SafeBoosC-III and the protocol, 2) cerebral NIRS monitoring, 3) SafeBoosC-III treatment guideline, 4) cranial ultrasound imaging and diagnosing of brain injury, and 5) GCP monitoring in SafeBoosC-III. All modules are designed as integrated training and certification modules, with each module consisting of a) learning material and b) a quiz. With the exception of the introduction module, all modules are built over a simplified adaptive framework, meaning that you are led directly to the quiz and will only be prompted to visit the learning materials if your answers to questions are wrong. If you answer all questions correctly, you have shown mastery of the subject matter and will be certified directly. As such the quiz is designed to recognize prior learning, as correct answers will get participants through the modules faster. For less experienced users, the option is given for the user to bypass the quiz and go directly to the learning materials first (Fig. 1).

**Fig. 2:**
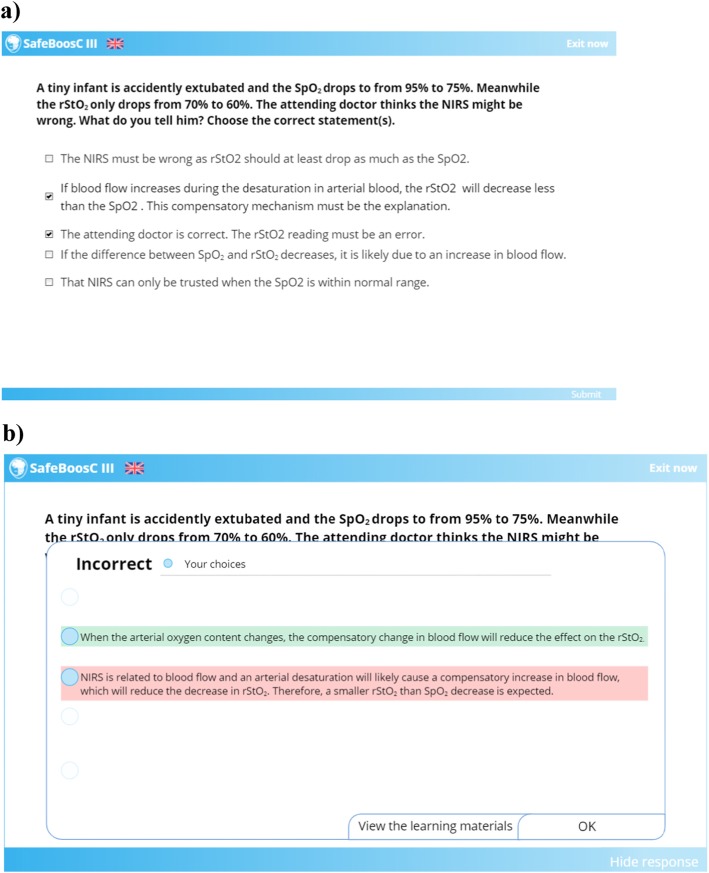
Example of a question from the NIRS module. **a** The case-text describing the clinical setting and the five answer possibilities; options two and three have been chosen. **b** Explanations to answers are presented; option two was correct, while option 3 was wrong. This means that the question is not passed and the participant will be presented with another question on the same learning objective and have to answer that correctly before completing the quiz

The content for all modules was developed with the same approach: Initially, a narrative text covering all essential knowledge on the subject was drafted. Based on the narrative, a number of learning objectives were developed, all clearly described according to Bloom’s Taxonomy’s cognitive domain [21] to specify which degree of mastery the user should show. The narrative was also used to write the learning material for each module. Next, two to four questions related to each learning objective were developed, thereby representing a pool of questions used to build the quiz. The questions strive to be as relevant as possible and were therefore formulated in a case-like manner, with a short description of a clinical situation given for each question, followed by a varying number of response options. Cases reflect clinical situations that could happen during the conduct of SafeBoosC-III. Often, there are several correct response options constituting the format of multiple-choice questions with several-of-many answers. To pass a question, all the correct answers and only correct answers must be ticked (Fig. 2a). Participants complete a module when they have answered one question per learning objective correctly. We could have chosen to require two (or three) correct answers per learning objective, but this would have inflated the volume of questions—and hence the costs—as well as the time to be used by participants. They will be exposed to new questions or re-exposed to questions they have already met from the quiz-pool on a continuous basis until the above criterion is met. Questions in the learning objectives that at any given time are not yet passed are presented in random order. This is done to reduce the risk that participants adopt a fast game, like a ‘trial and error’ strategy, rather than learning and understanding.

The teaching methodology is case-based and uses immediate detailed feedback, which means that participants will be presented with explanations for right and wrong answers as they go (Fig. 2b). This method has been shown to increase student performance in previous online medical education programs [22].

The complete web-based training and certification program will be hosted in a Moodle virtual learning environment (Moodle Pty Ltd, West Perth, WA, Australia), a commonly used shareware software within online medical training [10, 11, 23]. However, Moodle was not used as the platform for this pilot project due to restricted time. Instead, a direct link with immediate access to the module was used. The platform used in this pilot study was the Capital Region of Denmark’s primary platform for e-learning, providing almost 400 different training programs for 40,000 staff members (kursusportalen.plan2learn.dk).

### Training module on NIRS

The module on NIRS monitoring which was piloted in this study focuses on the principles of measuring cerebral oxygenation by NIRS, basic device operation, application and fixation of the sensor to the head of the infant, care of sensor and repositioning, the risk of skin marks, interpretation of measured values and the concept of venous-weighted tissue blood oxygenation. It consists of four learning objectives and 11 questions (Table 1). SHS and GG [24–28] took the lead in writing the learning material as well as questions for the quiz. MLH also participated in this process. MIR and SR programmed the training module in the interactive e-learning software Articulate Storyline (Articulate, New York, NY, US) and provided a direct URL link for participants to use. SR is an employee of the Copenhagen University Hospital e-learning section and is mainly responsible for programming all modules for the web-based training and certification program.

**Table 1:** Learning objectives and questions for the training module on NIRS monitoring

Learning objective	Question
Point out differences between NIRS tissue oxygenation (rStO_2_) and pulse oximetry	A father of a very preterm infant asks why the cerebral rStO_2_ is 65 when the SpO_2_ is 94. What do you tell him?
	A baby is pale and mottled and you suspect circulatory failure due to septic shock. You have a hard time getting a signal from the pulse oximeter, but the NIRS gives readings with no apparent problems. Choose the correct statement(s)
Recognise the consequences of rStO2 being a direct measure of cerebral oxygen consumption/supply balance and indirect measure of cardiac output	A tiny infant is accidentally extubated and the SpO_2_ drops from 95% to 75%. Meanwhile, the rStO_2_ only drops from 70% to 60%. The attending doctor thinks the NIRS might be wrong. What do you tell him?
	A colleague asks for help to understand what rStO2 really measures. Which of the following statements would you include in your explanation
	You care for a baby in the experimental group on the first day of life. Everything has been stable when the rStO2 alarm goes off and shows cerebral hypoxia. No other monitors are sounding an alarm. The ventilator runs normally, the SpO2 is stable around 92% and the mean arterial blood pressure is stable around 28 mmHg. As you look into the incubator to check the cerebral oximeter sensor you see that he has been bleeding from the umbilicus. It is a large spot on the linen and in the diaper and you estimate that the volume may be 10 ml. Could that be the explanation for the cerebral hypoxia?
Know the elements in starting up NIRS monitoring and interpret values during monitoring	You move the sensor to the other side of the forehead of a sick preterm infant as part of routine care. The parents notice that rStO_2_ is about 7 percentage points higher in the new position. What answers can you give them?
	rStO_2_ drops suddenly to 40%. What would you do? Please prioritise the following actions from first to last
	You have to start up monitoring cerebral oxygenation. Which of the following actions would you **not** do?
Know the side effects of NIRS monitoring	The parents ask if there are side effects to the near-infrared light used by the oximeter. Choose which statements you may include in your explanation
	You take over the care of a baby in the SafeBoosC trial. He is in the experimental group. Gestational age is 24 weeks and he is mechanically ventilated with high pressures and on high dose pressor (dopamine 15 microgram/kg/min) and yet the mean arterial blood pressure is only 24 mmHg. The situation, however, has been stable for the last 12 h. The cerebral oximeter seems to work well and the rStO2 is 65% (the hypoxic threshold of your oximeter is 58%). Choose what you will do?
	A mother notices a minor mark on the skin after you have moved the pulse oximeter sensor to another position. She is now concerned about the NIRS sensor as well. What answers can you give her?

Since SafeBoosC-III is a multinational trial, language barriers can pose as a challenge because the content of the web-based training program must be translated to all the languages and still hold an academic level which meets clinical standards without the translation process being too complex. Therefore, in order to test the feasibility of translating the content of the web-based training program and train staff members in local languages, the original English version was translated into both Spanish and Chinese. The translation was done locally by the national coordinators AP in Spain and GC in China, who conducted manual translation of the material from English to Spanish and Chinese, respectively. In China, GC conducted the translation with the aid of the online translation tool ‘youdao.com’ whenever he was in doubt of the correct translation. Due to limited resources, the quality and precision of the translations were not evaluated by external linguistic experts and no back-translation and comparison with the originals were conducted. Danish participants were trained in the English module and Chinese and Spanish participants in the Chinese and Spanish modules, respectively.

### Survey

Upon completion of the NIRS module, participants were asked to evaluate their experience by completing an online survey. For Spanish and Danish participants, the online survey was hosted in Google Analytics (Google LLC, Mountain View, CA, USA), but since Google is blocked in China, a Chinese survey program, Wenjuan (Shanghai Zhongyan Network, Shanghai, China), was used to host the Chinese survey. Participants completed the survey in local languages. As for the web-based training module, the translation of the survey was done locally by AP and GC. The online survey consisted of 15 closed-ended questions with answers on a three- or four-step Likert scale and seven open-ended questions with free-text answers. The structure and content of the closed-ended questions are based on Wang’s principles for e-learner satisfaction [29]. Open-ended questions were added to gain a deeper and more complex understanding of participants’ experiences and to clarify potential room for improvement. The survey covered the following themes: 1) performance, 2) learning material, 3) quiz material, 4) interface, and 5) preparation to use NIRS monitoring in a clinical context. MIR and MLH developed the online survey.

### Data analysis

Quantitative data from closed-ended questions were analysed using basic descriptive statistics. Analysis of answers to the open-ended questions followed the principles of thematic analysis as described by Braun and Clarke [30]. With an inductive and data-driven approach, an iterative six-step analysis was conducted to identify themes across the entire data set [30]. Initially, answers were systematically reviewed and coded. In total, 111 answers were coded into 70 codes, which subsequently were narrowed into 64 codes, based on their similarities (Table 2).

**Table 2:** Examples of data extract coding. Narrative to the left and codes to the right

Fine academic level, but some of the questions did not match the introduction material, which was a shame and frustrating (in relation to agreeing/strongly agree that the academic level of the quiz was appropriate)	1. Discrepancy between introduction material and quiz 2. Frustrations 3. Academic level appropriate 4. Introduction material insufficient to answer quiz questions
The question is not related to the learning material. The language is not enough concise and clear	1. Discrepancy between the introduction material and quiz 2. Unprecise language

All 64 codes were collated and grouped into seven candidate themes. In order to get a better overview of data, candidate themes were illustrated in mind maps and reviewed in relation to 1) their specific data extracts and 2) across the entire data set. In this process, themes that covered similar aspects were merged, and irrelevant themes were either deleted or re-assembled, which resulted in four final themes.

### Ethics approval and consent to participate

According to Danish, Chinese and Spanish laws, survey studies are not considered biomedical research and ethics approval was not therefore required to conduct this study. An information sheet written by MLH and GG explaining the purpose of the pilot project, that no personal data were collected and that all survey answers were recorded and analysed anonymously was distributed to the responsible investigators in each of the five participating neonatal intensive care units (AP, GC, MLH, XX and YZ). The five investigators invited relevant staff members to participate in the pilot study and, based on the information sheet, informed them of the study and data handling. All staff members had the possibility to ask the responsible investigators questions on the study and the possibility to decline participation in this pilot study. Since no personal identifiers were registered on participants, it was impossible to identify the identity of individual survey responders and thus withdrawal of data was not possible. This design was chosen to protect the participants against employers and responsible investigators back-tracking the performance or survey completion of individuals.

## Results

In total, 81 of 100 invited staff members (81%) entered the training module and completed the online survey. Fifty (62%), 16 (20%) and 15 (18%) staff members responded from China, Spain and Denmark, respectively. Of the 81 responders, 41 were doctors (51%) and 40 were nurses (49%). Previous experience with NIRS monitoring was reported by 46 of the 81 responders (57%), including 26 doctors (57%) and 20 nurses (43%) (Table 5). In Denmark, six of the 15 responders had previous experience (40%), in China 25 of 50 (50%), and in Spain 15 of 16 (94%).

### Closed-ended questions

#### Performance

Overall, responders spent a median time of 15 min (range 1 to 420 min) and a median number of seven questions (range 4 to 50 questions) to complete the NIRS module. Spanish responders were faster than both Danish and Chinese (median 10, 14 and 20 min, respectively) and used fewer questions to pass (median 4, 7 and 8, respectively) (Table 3). Doctors were faster than nurses (median 13.5 versus 20 min) and used fewer questions to pass (median 6 versus 9 questions) (Table 4). Responders with NIRS experience were faster than non-experienced (median 13.5 min versus 20 min) and spent fewer questions to pass (median 5.5 versus 8 questions) (Table 5).

**Table 3:** Time used and number of quiz questions used to complete the module and number of responding participants who answered either ‘agree’/‘strongly agree’ or ‘appropriate’ to the questions regarding the design of the module (data stratified by country)

Question	Denmark	Spain	China	Total
**Performance**				
Minutes to complete module, median [range]	14 [7–30] (11/15)^c^	10 [1–60] (13/16)^c^	20 [2–420] (46/50)^c^	15 [1–420] (70/81)^c^
Number of questions to complete module median [range]	7 [6–20] (5/15)^c^	4 [4–12] (13/16)^c^	8 [4–50] (43/50)^c^	7 [4–50] (61/81)^c^
**Learning material**				
Academic level of learning material appropriate, n/N (%)	14/15 (93)	15/16 (94)	40/50 (80)	69/81 (85)
Learning material sufficient to complete quiz^a^, n/N (%)	3/12 (25)	13/16 (81)	39/50 (78)	55/78 (70)
**Quiz**				
Academic level of quiz appropriate^a^, n/N (%)	14/15 (93)	15/16 (94)	46/50 (92)	75/81 (93)
Number of answering possibilities per question appropriate, n/N (%)	6/15 (40)	9/16 (56)	34/50 (68)	49/81 (60)
Quiz questions clinically relevant and up-to-date^a^	13/14 (93)	15/16 94)	49/50 (98)	77/80 (96)
**Interface**				
The NIRS module was stable and did not crash^b^, n/N (%)	6/15 (40)	9/15 (60)	42/50 (84)	57/80 (71)
**Preparation for using NIRS**				
Relevant to prepare for using the NIRS device^a^	13/15 (87)	12/15 (80)	47/50 (94)	72/80 (90)

^a^Pooling of the answers agree or strongly agree

^b^Yes to the statement

^c^ Number of responders answering the specific question and the total number of overall responders completing the online survey

**Table 4:** Time used and number of quiz questions used to complete the module and number of participants who answered either ‘agree’/‘strongly agree’ or ‘appropriate’ to the questions regarding the design of the module (data stratified by participants’ profession)

Question	Doctors	Nurses	Total
Performance			
Minutes to complete module, median [range]	13.5 [2–420] (34/41)^c^	20 [1–420] (36/40)^c^	15 [1–420] (70/81)^c^
Number of questions to complete module, median [range]	6 [4–30] (28/41)^c^	9 [4–50] (33/40)^c^	7 [4–50] (61/81)^c^
Learning material			
Academic level of learning material appropriate, n/N (%)	37/41 (90)	32/40 (80)	69/81 (85)
Learning material sufficient to complete quiz^a^, n/N (%)	28/38 (74)	27/40 (68)	55/78 (70)
Quiz			
Academic level of quiz appropriate^a^, n/N (%)	38/41 (93)	37/40 (93)	75/81 (93)
Number of answering possibilities per question appropriate, n/N (%)	29/41 (71)	20/40 (50)	49/81 (60)
Quiz questions clinically relevant and up-to-date^a^	39/40 (98)	38/40 (95)	77/80 (96)
Interface			
The NIRS module was stable and did not crash^b^, n/N (%)	29/41 (71)	28/39 (72)	57/80 (71)
Preparation for using NIRS			
Relevant to prepare for using the NIRS device^a^	35/40 (88)	37/40 (93)	72/80 (90)

^a^Pooling of the answers agree or strongly agree

^b^Yes to the statement

^c^ Number of responders answering the specific question and the total number of overall responders completing the online survey

**Table 5:** Time used and number of quiz questions used to complete the module and number of participants who answered either ‘agree’/‘strongly agree’ or ‘appropriate’ to the questions regarding the design of the module (data stratified by participants’ previous experience with NIRS monitoring)

Question	Experience	No experience	Total
Performance			
Minutes to complete module, median [range] (n/N)	13.5 [1–420] (40/46)^c^	20 [4–420] (30/35)^c^	15 [1–420] (70/81)^c^
Number of questions to complete module, median [range] (n/N)	5.5 [4–20] (36/46)^c^	8 [4–50] (25/35)^c^	7 [4–50] (61/81)^c^
Learning material			
Academic level of learning material appropriate, n/N (%)	39/46 (85)	30/35 (86)	69/81 (85)
Learning material sufficient to complete quiz^a^, n/N (%)	32/44 (73)	23/34 (68)	55/78 (70)
Quiz			
Academic level of quiz appropriate^a^, n/N (%)	41/46 (89)	34/35 (97)	75/81 (93)
Number of answering possibilities per question appropriate, n/N (%)	27/46 (59)	22/35 (63)	49/81 (60)
Quiz questions clinically relevant and up-to-date^a^	43/46 (93)	34/34 (100)	77/80 (96)
Interface			
The NIRS module was stable and did not crash^b^, n/N (%)	34/45 (76)	23/35 (66)	57/80 (71)
Preparation for using NIRS			
Relevant to prepare for using the NIRS device^a^	41/45 (91)	31/35 (89)	72/80 (90)

^a^Pooling of the answers agree or strongly agree

^b^Yes to the statement

^c^ Number of responders answering the specific question and the total number of overall responders completing the online survey

#### Learning material

Overall, 69 of 81 (85%) responders found the academic level of the learning material appropriate and none found it too easy. Of the 12 responders who found the learning material too advanced, ten were from China, one was from Denmark and one was from Spain (Table 3). Eight of 40 (20%) nurses found the learning material too advanced compared to four of 41 (10%) doctors (Table 4). Additionally, no relevant difference was seen between responders experienced in NIRS monitoring and those with no experience (seven of 46 (15%) experienced versus five of 35 non-experienced (14%)) (Table 5). When asked if the introduction material was sufficient to answer quiz questions, 23 of 78 (29%) responders disagreed or strongly disagreed, nine from Denmark, 11 from China and three from Spain (Table 3). More nurses (13 of 41 (32%)) than doctors (10 of 38 (26%)) disagreed or strongly disagreed with this statement (Table 4). Amongst those with NIRS experience, 12 of 44 (27%) disagreed or strongly disagreed compared to 11 of 34 (32%) responders with no previous experience (Table 5).

#### Quiz

Seventy-five of 81 (93%) responders agreed or strongly agreed that the academic level of questions was appropriate. Of those who disagreed or strongly disagreed, no relevant difference was found between countries (Table 3) or clinical positions (Table 4). Of the six disagreeing or strongly disagreeing on the statement, five were experienced in NIRS monitoring (Table 5). Thirty-two of 81 (40%) responders thought there were too many answer possibilities for each question, primarily nurses (20 of 40 (50%) nurses compared to 12 of 41 (29%) doctors) (Table 4) and Danish responders (nine of 15 (60%) compared to seven of 16 (43%) Spanish and 16 of 50 (32%) Chinese responders) (Table 3). Among those with NIRS experience, 19 of 46 (41%) thought there were too many answer possibilities compared to 13 of 35 (37%) with no experience (Table 5). When asked if the quiz questions were clinically relevant and up-to-date, 77 of 80 (96%) responders agreed or strongly agreed on this.

#### Interface

Almost one-third of all responders (23 of 80 (29%)) experienced a crash once or multiple times while accessing the NIRS module . It seemed that the problem was greatest in Denmark and Spain where nine of 15 (60%) and six of 15 (40%) reported experiencing a crash, compared to only 8 of 50 (16%) in China (Table 3). Among doctors and nurses, 12 of 41 (29%) and 11 of 39 (28%) experienced a crash, respectively (Table 4). Eleven of 45 (24%) experienced with NIRS and 12 of 35 (34%) non-experienced responders reported a crash (Table 5).

#### Preparation for using NIRS

When asked if the module was relevant to prepare staff members to use the NIRS device, 72 of 80 (90%) agreed or strongly agreed on this (13 of 15 (87%) Danish, 12 of 15 (80%) Spanish and 47 of 50 (94%) Chinese responders) (Table 3). No relevant difference was seen between clinical positions (35 of 40 (88%) doctors and 37 of 40 nurses (93%)) or between experience levels (41 of 45 (91%) experienced and 31 of 35 (86%) non-experienced) (Tables 4 and 5).

### Open-ended questions

**Fig. 3:**
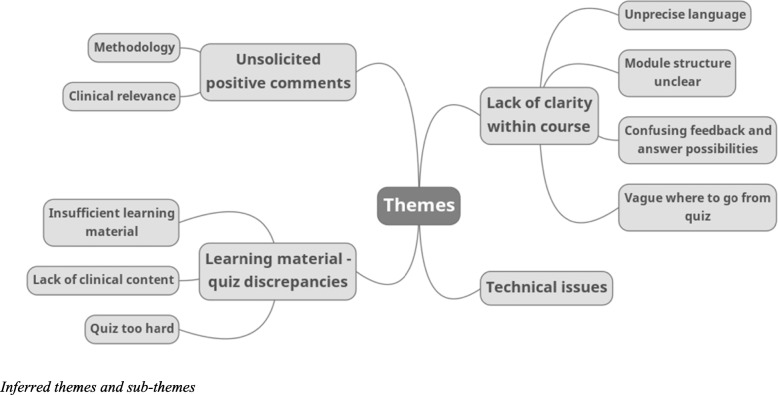
Inferred themes and sub-themes

The thematic analysis resulted in four essential themes, accompanied by sub-themes (Fig. 3). The themes were 1) learning material-quiz discrepancies, 2) lack of clarity within course, 3) technical issues and 4) unsolicited positive comments. These four themes elicit key concepts that are essential throughout the data.

#### Learning material—quiz discrepancies

Some responders (*n* = 18) described a discrepancy between the learning material and the quiz, with several stating that the learning material was insufficient to adequately answer the questions in the quiz:

“*For someone who know [s] little or nothing about the topic, the introduction material is not sufficient enough to answer the quiz questions*” *Doctor*

One responder stated that despite being committed and working hard to gain a comprehension of the learning material, they struggled with answering the questions correctly and finishing the course:

“*Put in a great effort to understand the intro material and I was surprised that I could not answer questions correctly. I did not feel that there was a connection between theory in the introduction material and questions*” *Nurse*

A few mentioned (*n* = 6) that the learning material was too simple or not detailed enough and was lacking comprehensiveness:

“*Additional knowledge is needed in the principles and concepts section*” *Nurse*

As a possible consequence of this discrepancy, some responders (*n* = 10) also expressed that the content of the course was too hard:

“*The content is too hard to understand*” *Nurse*

“*The questions are difficult, and the basic courses are few*” *Doctor*

Some responders (*n* = 23) also stated that specific clinical content was missing in the learning material, which made it difficult to complete the quiz. A specific concern raised (*n* = 14) was the absence of knowledge regarding the practicality behind the usage and handling of the NIRS device:

“*Risk of skin marks and side-effects is not described sufficiently in the introduction material*” *Doctor*

The lack of clinical content left a few responders (*n* = 4) feeling unequipped for answering questions in relation to this:

“*No introduction to how you prepared for NIRS monitoring, so it was pure guessing—you have no idea whether you need to calibrate/shave/wash or something else (prior monitoring), if you have not been told forehand*” *Nurse*

#### Lack of clarity within the course

Language issues were mentioned (n = 6), including that the language was not precise and clear, which made it hard to understand the context of the course. This was voiced by Spanish (*n* = 1) and Chinese (*n* = 5) participants:

“ *… The language is not enough concise and clear*” *Doctor*

The transparency of the module’s structure was also criticised, with a few responders (*n* = 7) stating that the feedback mechanism was hard to figure out:

“*[The module] did not tell me what my wrong answers I had, and therefore I didn’t know what the correct answers were and I couldn’t find it in the introduction material*” *Nurse*

In this event, one mentioned that it was hard to learn something from answering incorrectly:

“*It would be nice if one could learn something by answering wrong, hence that you could use the box that pops up after you answer incorrectly to see what was the correct answer.*” *Nurse*

In specific regards to the module lacking clarity, the deficient explanation of the quiz set-up was described. One responder expressed that it should be stated more explicitly how the module was structured:

“*Very good, but I was not prepared for a case-setup—and many answer possibilities were not mentioned in the introduction material*” *Nurse*

A few (*n* = 4) respondents stated that having multiple answer possibilities was an issue:

“*I think the quality of learning is increased if there are more questions with fewer answer possibilities. The purpose is learning and I think this could be heightened if one is presented with more questions with lesser answer possibilities … .*” *Doctor*

#### Technical issues

Technical issues seemed to be a source of frustration in this course. Responders answered that the module entered into a loop of incorrect questions (*n* = 4), that it crashed (*n* = 8), that the speed was slow (*n* = 5), and that the screen froze (*n* = 12), with one responder describing how it froze three to four times in a row, which caused this person to restart and begin all over again:

“*If you do it, you will be stuck, you can not finish it, what the hell*” *Doctor*

“*The page hangs on some occasions and does not allow to advance. When there is an incorrect answer, it loops in and you must restart the questionnaire to get out of there*” *Doctor*

The accessibility also seemed to be a problem. A few (*n* = 4) experienced that they could not easily navigate between the quiz and learning material without losing answers or facing a module crash, which in some cases led to a failure to finish the quiz:

“*Problems when some question is incorrect: it does not allow one to advance, in spite of reviewing the material and you must leave the page*” *Doctor*

#### Unsolicited positive comments

Despite the open-ended questions being focused on clarifying any critique points of the module as well as potential improvements, some responders (*n* = 12) also commented on the positive aspects of the module. Some applauded the clinical relevancy and fitness for clinical use:

“*Suitable for application of clinical*” *Doctor*

Others were positive towards the method of learning:

“*I really like the methodology in this e-learning course …* ” *Doctor*

Some were also generally positive such as:

“*Just right, very good*” *Doctor*

“*Super topic*” *nurse*

“*Very helpful*” *nurse*

“*Relatively friendly*” *nurse*

## Discussion

This pilot study of a module on cerebral NIRS monitoring for the SafeBoosC-III web-based training and certification program shows that it is possible to complete the module within a reasonable time frame, that the academic level is appropriate and that clinical relevance is high, irrespective of previous experience, clinical position or nationality.

In order to prepare for practical use, training must include clinically relevant scenarios. In the SafeBoosC web-based training and certification program, training cases are based on real-life scenarios and written by clinically experienced neonatologists and experts in the field [24, 25, 31–33], thereby making it possible to merge wide clinical experience and up-to-date literature within the field.

The external validity of our results is high [34] since the training module was tested in three different countries (Denmark, Spain and China), across two continents (Europe and Asia). Furthermore, we invited participants both with- and without previous experience on NIRS monitoring to participate. This was done to evaluate whether previous experience affected performance and comprehension. In SafeBoosC-III, the level of NIRS experience will vary between departments; thus, knowledge on feasibility of the certification and training program dependent on previous experience level is important. By using both closed- and open-ended questions, we were able to gain a wider and deeper understanding of the participants’ experience, which revealed important strengths and limitations of our design.

Translation of the training module was done manually by AP and GC (see “Methods” section) without any external translation support. Due to limited resources, we were not able to assess the quality and precision of the translations from English to Spanish and Chinese and were therefore not able to determine whether the quality and precision of the translations affected the difference in performance parameters and satisfaction rates. Despite a reasonable participation rate with 81 of 100 participants completing the online survey, we do not know for certain if all 81 responders completed the module. When looking at performance data (time to completion and number of questions to completion), 77 of the 81 responders had entered data for at least one of these parameters. However, two of the 77 responders commented that they did not complete the module, despite entering data on performance. Thus, we do not find data entry on performance parameters reliable as a measure of module completion. If we ought to rely on comments from responders, a total of five commented that they did not complete the module, primarily due to technical issues. Furthermore, we do not know whether the 19 participants who did not answer the survey still entered the training module but, due to unknown reasons, refrained from participating in the survey. Theoretically, it is possible that some of the 19 participants have been training in the module but gave up before completion and therefore did not answer the survey. Due to restricted time, it was not possible for us to host the piloting in Moodle, which would have made it possible to track completion rates.

When looking at performance data, the ranges of estimates are wide, with an upper limit of 50 questions for ‘number of questions to completion’ and 420 min for ‘time to completion’ (Table 3). However, only one responder answered 50 questions, one answered 30 questions and the remaining 79 responders answered using 20 questions or less. Regarding ‘time to completion’, six responders reported that they spent 420 min in the module, but only between 20 and 8 questions. They were all from the same country. The module automatically tracks time spent in the module, and when you reach completion, it will report the total time until completion. We suspect, therefore, that the six responders have had the module open throughout a 7-hour period but only trained part of the time. The remaining 75 responders spent 60 min or less.

Despite that web-based training provides a platform to train large numbers of staff across multiple countries, it also has the disadvantage of not knowing exactly how training was conducted locally. Since we could not monitor training on-site, we do not know whether responders trained in groups instead of individually, or how much they supported each other in completing the module. This could potentially affect performance data as well as answers to the questionnaire, thereby decreasing the validity of our results. However, using the SafeBoosC web-based training and certification program for group training instead of individual training may also be the case outside this pilot study; thus, this might depict how training and certification will be conducted when it is implemented externally. The module is structured and built for individual training, but group training may encourage discussions regarding the learning topics and therefore an increased learning opportunity.

By evaluating participants’ experience only through an online survey, we have potentially missed out important information and thereby the foundation for additional improvement. A semi-structured interview [35] with randomly selected responders from each country might have given us a deeper understanding of responses. However, due to restricted time and limited budget, this was not possible to conduct.

Despite positive responses, this piloting also revealed room for improvement, as described in the “Results” section. Major critique points included 1) too many answer possibilities, 2) inadequate correlation between learning material and quiz, and 3) too many technical problems hindering completion of the module. All of these points have been taken into consideration when we revised this specific module as well as designed the additional modules for the SafeBoosC-III web-based training and certification program; at first, we revised and edited all question within the modules so that only one or two correct answer possibilities persisted, in contrast to the previous design where some questions held up to five correct answers. We also scaled the questions so that the maximum number of answer possibilities was limited to five, as opposed to previously having up to ten possibilities. This condensing of the questions required that we split some of the complex questions into two or more focused questions, thereby creating a larger pool of questions per learning objective. Secondly, for each module, we cross-checked the content of the learning material with questions in the quiz in order to identify inconsistencies. If such were found, relevant content was added to the learning material in order to ensure adequate coverage of the learning material.

Regarding the technical problems, a new IT consultant identified several errors in the coding that caused crashes and other technical difficulties. These were corrected and implemented in all the training modules.

As of today, the full-scale SafeBoosC web-based training and certification program is hosted in Moodle on a commercial platform and up-and-running. It has been translated into Chinese, Turkish, Spanish, German and French using a similar approach as was done for this pilot study. So far, more than 500 staff members have trained, and we have not yet received complaints related to any of the major critique points revealed in the pilot phase, including technical errors.

We hope that our reporting of developing and implementing web-based training for a pragmatic, multinational study will encourage other trialists to take a similar approach despite limited funding, as well as reporting on the process for the benefit of peers.

## Conclusion

We believe that we provide evidence of the feasibility of developing a multilingual web-based training program for an international trial, despite challenges such as low budget, language barriers and possibly differences in the clinical training of staff. Exploring the integration of training and certification for international trials, the positive results of this study motivate further developments.

## Data Availability

The datasets used and/or analysed during the current study are available from the corresponding author on reasonable request.

## Abbreviations

GCP: Good Clinical Practice
NIRS: Near-infrared spectroscopy
SafeBoosC: Safeguarding the brains of our smallest children
USD: United States dollars
